# Atypical presentation of colorectal carcinoma with sole multiple osteolytic bone metastases: a case report

**DOI:** 10.1186/s13256-021-02795-5

**Published:** 2021-05-21

**Authors:** Ren Kawamura, Yudai Suzuki, Yukinori Harada, Taro Shimizu

**Affiliations:** Department of Diagnostic and Generalist Medicine, Dokkyo Medical University Hospital, Mibu, Tochigi 321-0293 Japan

**Keywords:** Colorectal cancer, Osteolytic lesions, Bone biopsy, Diagnostic delay

## Abstract

**Background:**

The incidence of colorectal cancer in persons aged < 50 years has been increasing. The diagnosis of colorectal cancer is not difficult if the patient has typical symptoms; however, diagnosis may be difficult in cases with atypical symptoms and signs. We present here an atypical case of metastatic colorectal cancer with fever and sudden onset paraplegia as the sole manifestations. The patient had multiple osteolytic lesions without gastrointestinal symptoms or signs, which resulted in a diagnostic delay of colorectal cancer.

**Case presentation:**

A 46-year-old Japanese man was transferred to our hospital for evaluation of fever. He had developed fever 8 weeks previously and had been first admitted to another hospital 5 weeks ago. The patient was initially placed on antibiotics based on the suspicion of a bacterial infection. During the hospital stay, the patient experienced a sudden onset of paralysis and numbness in his both legs. Magnetic resonance imaging showed an epidural mass at the level of Th11, and the patient underwent a laminectomy. Epidural abscess and vertebral osteomyelitis were suspected, and antimicrobial treatment was continued. However, his fever persisted, and he was transferred to our hospital. Chest, abdominal, and pelvic computed tomography (CT) with contrast showed diffusely distributed osteolytic lesions. Fluorodeoxyglucose-positron-emission tomography showed high fluorodeoxyglucose accumulation in multiple discrete bone structures; however, no significant accumulation was observed in the solid organs or lymph nodes. A CT-guided bone biopsy obtained from the left iliac bone confirmed the evidence of metastatic adenocarcinoma based on immunohistochemistry. A subsequent colonoscopy showed a Borrmann type II tumor in the sigmoid colon, which was confirmed to be a poorly differentiated adenocarcinoma. As a result of shared decision-making, the patient chose palliative care.

**Conclusions:**

Although rare, osteolytic bone metastases as the sole manifestation can occur in patients with colorectal cancer. In patients with conditions difficult to diagnose, physicians should prioritize the necessary tests based on differential diagnoses by analytical clinical reasoning, taking into consideration the patient’s clinical manifestation and the disease epidemiology. Bone biopsies are usually needed in patients only with sole osteolytic bone lesions; however, other rapid and useful non-invasive diagnostic tests can be also useful for narrowing the differential diagnosis.

## Introduction

Globally, the prevalence of colorectal cancer is increasing [1]. Although colorectal cancer has a relatively high cure rate, it remains one of the major causes of cancer-related death worldwide [2]. Moreover, the incidence of colorectal cancer has been increasing in the general population under the age of 50 years. In these younger patients with colorectal cancer, delayed diagnosis due to physician and patient delay because of their atypical presentation with non-specific symptoms has been raised as a major problem [3].

Bone metastasis is common and is one of the major prognostic factors in patients with advanced colorectal cancer. The prognosis of patients with bone metastases at the time of the diagnosis of colorectal cancer has been reported to be very poor, even among patients with advanced colorectal cancer [4]. Since bisphosphonate use has been shown to improve prognosis by 2 months even in these patients, timely diagnosis is essential [4]. However, timely diagnosis can be challenging when patients with colorectal cancer present with an atypical manifestation, such as bone metastasis only [5–7].

Consequently, considering colorectal cancer as the differential diagnosis can be tricky when patients who are relatively young for the disease present only with bone lesions without any gastrointestinal symptoms or signs. Furthermore, paraplegia has rarely been reported as the triggering symptom for the diagnosis of colorectal cancer [7]. Here, we describe the case of a 46-year-old man who presented only with fever and sudden onset paraplegia, who was ultimately diagnosed with colorectal cancer with bone metastases only.

## Case presentation

**Fig. 1:**
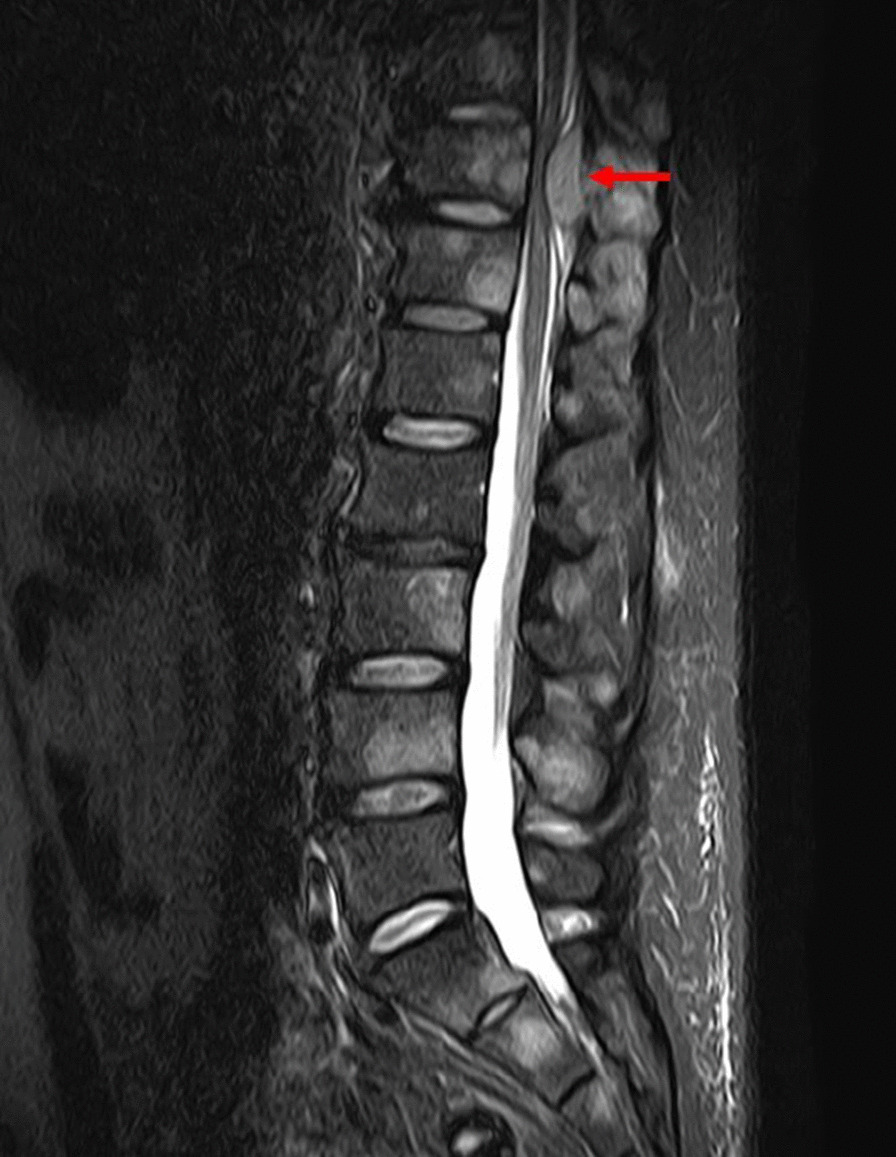
Magnetic resonance imaging of the lumbar spine showing an epidural mass at the level of thoracic vertebra 11 (Th11; red arrow) and multiple high-intensity vertebral lesions on short-time inversion recovery imaging

A 46-year-old Japanese man was transferred to our hospital for the evaluation of fever. He had a history of hypertension and no history of mental illness. There was no family history of malignancy or hematological disease. He had a 20 pack-year smoking history. He had been working as an engineer at an electronics company before he was hospitalized. The patient had developed fever 8 weeks prior to being admitted to our hospital. At that time, he was anorexic, but denied nausea, vomiting, diarrhea, constipation, bloody stool, or weight loss. He was first admitted to another hospital for the evaluation of persistent fever 5 weeks prior where he was initially treated with antibiotics (sulbactam/ampicillin, followed by ceftriaxone) based on the suspicion of a bacterial infection. However, the antibiotics were discontinued because no bacteria grew on the blood, sputum, and urine culture. During this period, he experienced a sudden onset of paralysis and numbness in his both legs. Magnetic resonance imaging (MRI) showed an epidural mass at the level of the eleventh thoracic vertebra (Th11) (Fig. 1), and the patient underwent a laminectomy at the levels of Th10–12. Epidural abscess and vertebral osteomyelitis were suspected based on the intraoperative visual findings, even though the bacterial cultures of intraoperative specimens were negative. No tissue was sent for histopathological examination. Antimicrobial treatment (meropenem and vancomycin) was restarted; however, his fever persisted, and he was then transferred to our hospital.

**Fig. 2:**
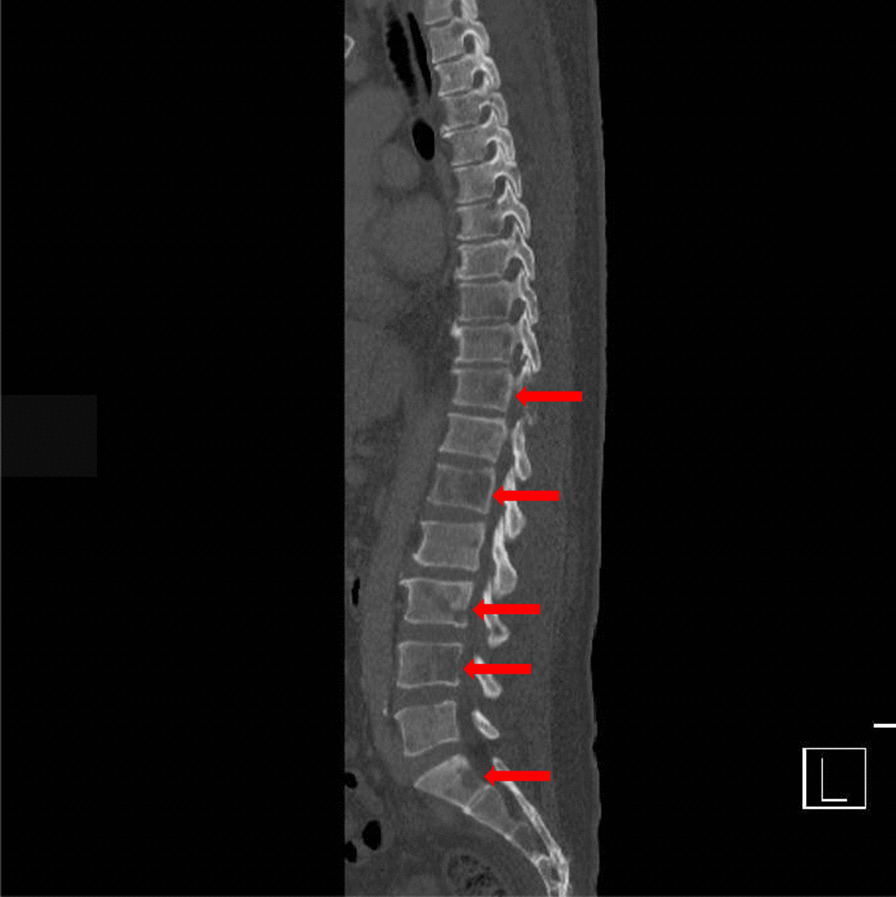
Computed tomography image showing multiple osteolytic lesions throughout the spine (red arrows)

At the time of admission to our hospital, his Eastern Cooperative Oncology Group performance status was 4. The patient reported fecal and urinary incontinence, but denied nausea, vomiting, diarrhea, constipation, bloody stool, or weight loss. His vital signs included a temperature of 38.2 ℃, blood pressure of 119/62 mmHg, heart rate of 109 bpm, respiratory rate of 30 breaths per minute, and oxygen saturation at 90% on ambient air. There was no tenderness on the back. Paraplegia and numbness were detected below the level of the first lumbar vertebra (L1). The laboratory test results (Table 1) showed normocytic anemia and elevated aspartate transaminase, alanine transaminase, alkaline phosphatase, lactate dehydrogenase, C-reactive protein, and ferritin levels. The level of prostate-specific antigen was within the normal range. There was no evidence of paraproteinemia. Chest, abdominal, and pelvic computed tomography (CT) with contrast showed diffusely distributed osteolytic lesions (Fig. 2) and a left adrenal adenoma, but no other abnormal findings were detected. There was no abnormal wall thickening of the colon. A thoracic and lumbar spine MRI showed high-intensity lesions in the diffusion-weighted image, and short-time inversion recovery imaging and low-intensity lesions on T1 weighted from second cervical vertebra (C2) to L5.

**Table 1.** Laboratory test results at the first visit

Laboratory tests	Values
White blood cell count	18.6 × 10^9^/L
Hemoglobin	102 g/L
Platelet count	340 × 10^9^/L
Aspartate aminotransferase	146 U/L
Alanine aminotransferase	116 U/L
Lactate dehydrogenase	5571 U/L
Alkaline phosphatase	7830 U/L
Gamma glutamyl transferase	108 U/L
Blood urea nitrogen	58 mg/dL
Creatinine	1.3 mg/dL
Calcium	9.2 mg/dL
Creatine kinase	1103 U/L
C-reactive protein	> 40 mg/L
Ferritin	50,815 ng/mL
Prostate-specific antigen	0.623 ng/mL

Based on the presence of multiple osteolytic lesions, differential diagnoses at this time included hematological malignancies (lymphoma, leukemia, and multiple myeloma), metastatic carcinoma, *Mycobacterium tuberculosis* infection, primary hyperparathyroidism, and primary amyloidosis. A specific type of lymphoma, which was characterized by the absence of enlarged lymph nodes, such as intravascular lymphoma, remained a diagnostic possibility. Based on the laboratory and imaging findings, multiple myeloma, primary amyloidosis, leukemia, and hyperparathyroidism were unlikely. Given that the patient did not have an apparent history of exposure and his blood and spinal fluid mycobacterial cultures and interferon-gamma release assays were negative, tuberculosis also seemed to be less likely. Therefore, the physicians considered intravascular lymphoma and metastatic carcinoma as the most likely differential diagnoses. In particular, intravascular lymphoma was suspected based on the fever of unknown origin with the poor general condition, high lactate dehydrogenase level, and osteolytic lesions throughout the body with no identifiable mass lesions or lymphadenopathy.

**Fig. 3:**
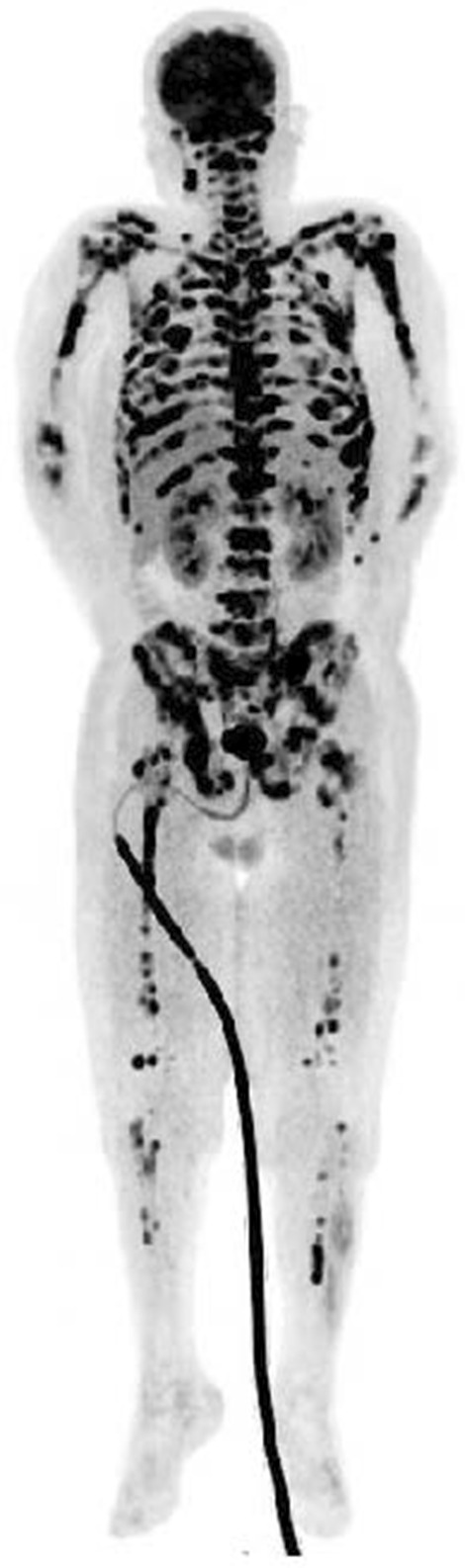
Fluorodeoxyglucose-positron-emission tomography showing high fluorodeoxyglucose accumulations in multiple discrete bone structures and mild accumulation in the left adrenal gland, but no significant accumulation observed in solid organs or lymph nodes

**Fig. 4:**
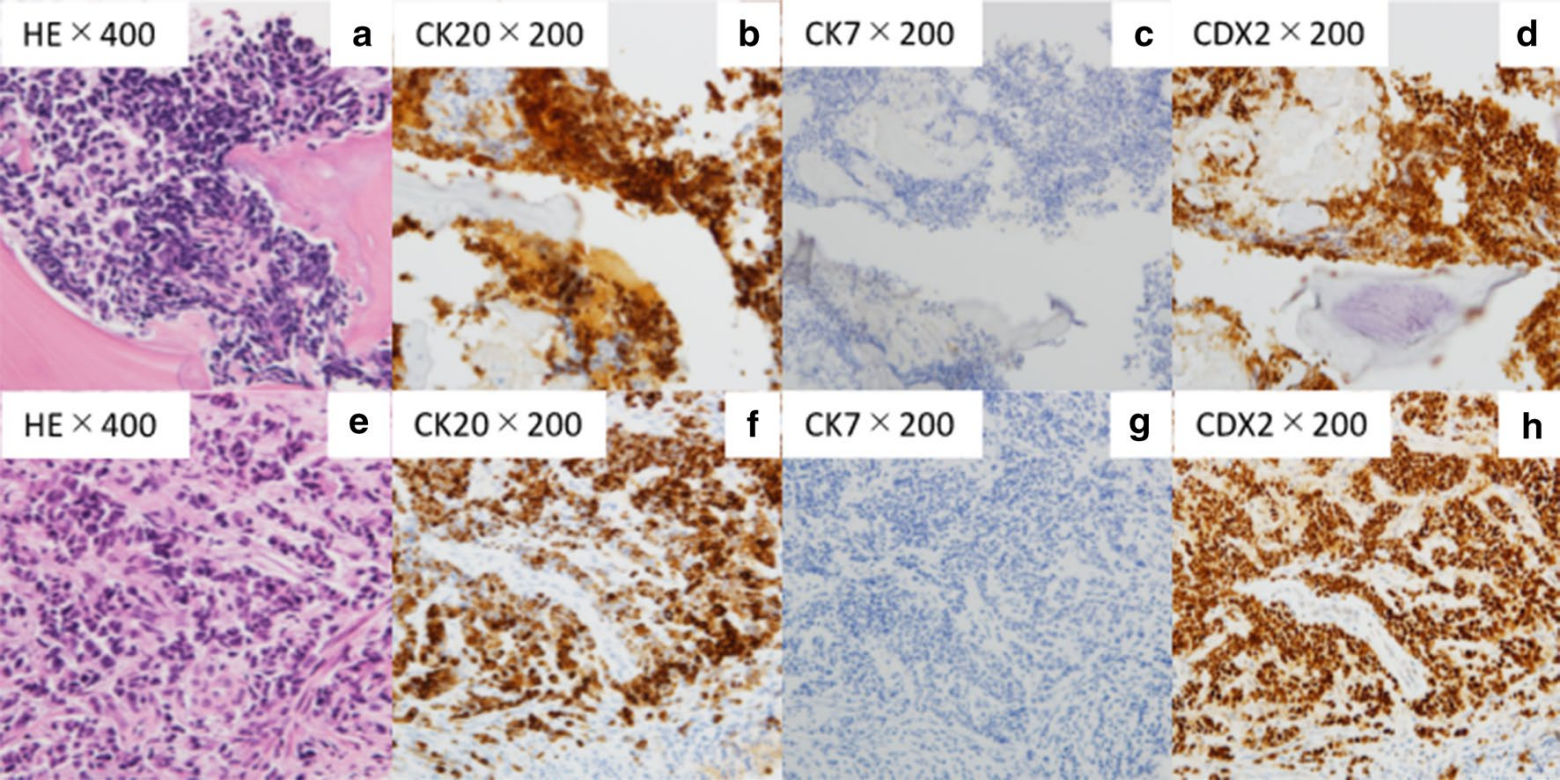
Histopathological findings of the left iliac bone (**a**–**d**) and the sigmoid colon tumor (**e**–**h**). Both the bone and the colon tumor were positive for CK20 and CDX2 immunostaining but negative for CK7 immunostaining.* HE* hematoxylin and eosin

**Fig. 5:**
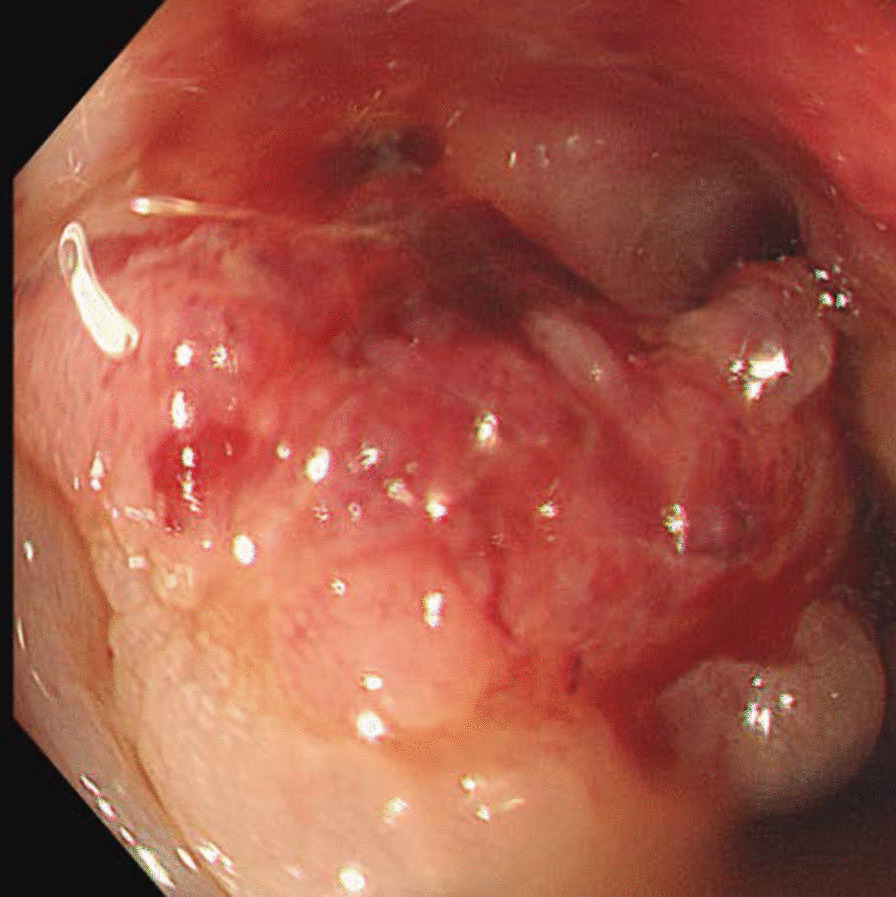
Colonoscopy image. The colonoscopy revealed a Borrmann type II tumor with diameter of more than 20 mm in the sigmoid colon

A random skin biopsy revealed no abnormalities, and a bone marrow biopsy from the left iliac bone revealed no contributory findings. Fluorodeoxyglucose-positron-emission tomography (FDG-PET) showed high FDG accumulation in multiple discrete bone structures, including the left iliac bone, and mild accumulation in the left adrenal gland, which was likely due to the adrenal adenoma; however, no significant accumulation was observed in solid organs, intestines, or lymph nodes (Fig. 3). A CT-guided bone biopsy taken from the left iliac bone, where FDG had accumulated the most, confirmed the evidence of metastatic adenocarcinoma based on immunohistochemistry (Fig. 4a–d). Colorectal cancer was suspected based on the results that the fecal occult blood test was positive and the carcinoembryonic antigen (CEA) submitted later was high at 2260 ng/mL. Alpha-fetoprotein and carbohydrate antigen 19-9 (CA19-9) levels were normal. A subsequent colonoscopy showed a Borrmann type II tumor with a diameter of more than 20 mm in the sigmoid colon (Fig. 5), which was confirmed by histopathology to be a poorly differentiated adenocarcinoma. The immunohistochemistry pattern of the tumor was the same as that of the bone lesion (Figure 4e–h). The patient was finally diagnosed with advanced colorectal carcinoma with multiple bone metastases. It took 1 month from admission to our hospital to the definitive diagnosis. His fever persisted during the investigation, but resolved with naproxen. After a shared decision-making, the patient chose palliative care without chemotherapy. He returned to the previous hospital, where he received palliative care, and died 4 weeks later.

## Discussion

A diagnostic delay occurred in this case. The delay was attributed to two factors: (1) the uncommon presentation of the disease and (2) the inadequate ordering of diagnostic tests.

Colorectal cancer metastasis to the bone is common, with a prevalence of 2–24%. However, bone metastasis as the sole manifestation of colorectal cancer is rarely observed and occurs in only 1–2% of these patients [5–7]. In terms of the metastatic bone pattern, bone metastases of colorectal cancer usually disseminate to multiple sites, with spinal metastasis being the most common, followed by metastasis to the pelvis and long bones [4, 5]. While patients with colorectal cancer metastasis to the bone may develop bone pain, fracture, spinal cord compression, and hypercalcemia, > 60% of patients can be asymptomatic, with bone pain possibly occurring in only 25% of patients [5]. Colorectal cancer metastasis to the bone can be difficult to detect, largely due to the rarity of bone metastases as the sole manifestation of colorectal cancer, and many patients with such metastases are asymptomatic [5]. Furthermore, to the best of our knowledge, paraplegia, as manifested in this patient, has rarely been reported as a triggering symptom for the diagnosis of colorectal cancer [7]. A range of symptoms, including fever and paraplegia without gastrointestinal symptoms, are rare presentations in patients with colorectal cancer. In the case presented here, these unusual presentation patterns clouded the physicians’ judgments in terms of suspecting colorectal cancer as a differential diagnosis. However, based on the evidence that the incidence of colorectal cancer in the general population under the age of 50 years has been increasing and that these patients often present to hospitals later in advanced stages and with atypical symptoms [3], physicians should consider colorectal cancer as the primary tumor in patients with suspicious findings of bone metastases of malignant tumors even if they are under the age of 50 years.

In this case, the multiple imaging studies revealed no abnormal findings other than bone lesions, leading the physicians to consider intravascular lymphoma as a possible diagnosis, as opposed to metastatic carcinoma. This diagnostic reasoning resulted in the bone marrow and skin biopsies; however, there were no findings suggestive of lymphoma. The biopsies were related to the diagnostic delay because the physicians deferred additional testing while waiting for the results. In retrospect, the physicians should have adopted a basic approach and considered starting with minimally invasive tests that would be useful in determining the other differential diseases. When carcinoma in Japanese men in their 40s is being considered, the fact that colon cancer is one of the most common malignant tumors, apart from stomach and lung cancer, has to be taken into account [8]. Upper gastrointestinal endoscopy and colonoscopy are useful and less invasive tests for the detection of colorectal and gastric cancers. In this case, lung cancer was less likely because chest CT and PET-CT showed no evidence of abnormalities. CEA and CA19-9 have also been reported to be useful for distinguishing bone metastasis caused by carcinoma from primary bone tumors or hematological malignancy, even though these tumor markers do not identify the primary site of the tumor [9, 10]. Hence, less invasive tests, such as endoscopy, and measuring tumor markers could have been options for deciding the next evaluation while waiting for the biopsy results.

Conversely, repeated biopsies of suspicious areas are warranted, especially when findings on MRI or PET-CT are positive even if the initial examination is negative [11]. The initial bone marrow biopsy taken from the left iliac bone was negative in this case; however, FDG accumulation was observed in the same area of the biopsy site on the following PET-CT, and the second bone biopsy ultimately led to the diagnosis. In fact, given that the high sensitivity of CT-guided bone biopsy (96.7%) has been reported in the diagnosis of metastatic bone tumors, the second CT-guided biopsy seemed to play a key role in the correct diagnosis of the present case [11]. Regarding the bone biopsy, one can assume that in the present case the definitive diagnosis could have been made earlier if the tissue had been sent for histological examination at the time of the initial spinal surgery. We do not know exactly why the bone tissue or pus were not sent for histological study: however, it would appear that some cognitive biases, such as base rate neglect, for assessing the probability of malignancy and anchoring and confirmation bias kept the physicians stuck to a diagnosis of infection. To avoid such cognitive biases, physicians should utilize the analytical thinking process for assessing whether intuitive diagnosis is correct or not.

## Conclusion

This is a case of colorectal cancer with an uncommon presentation in that metastasis was only to the bones. Physicians should be aware that the incidence of colorectal cancer in general population under the age of 50 years has been increasing and that these patients often present in advanced stages with atypical manifestations, such as multiple bone metastases as the sole manifestation. Since CT-guided bone biopsy is invasive and sometimes time consuming, although it has a high sensitivity for the diagnosis of metastatic bone tumors, more rapid, useful, and less invasive diagnostic tests, such as endoscopy, should be applied first when attempting to diagnose patients presenting with multiple bone metastases. Physicians should always be cautious about cognitive biases, particularly when seeing patients with atypical presentations (Table 2).

**Table 2:** Learning points

Physicians should know that patients with advanced colorectal cancer under the age of 50 years can present with atypical manifestations, such as bone metastases as the sole manifestation.
Multiple osteolytic lesions often require bone biopsies for diagnosis, but less invasive appropriate diagnostic tests should be considered first during the diagnostic process.
Physicians should always be cautious about cognitive biases, particularly when seeing patients with atypical presentations.

## Data Availability

Not applicable.

## Abbreviations

CA19-9: Carbohydrate antigen 19-9
CEA: Carcinoembryonic antigen
CT: Computed tomography
FDG-PET: Fluorodeoxyglucose-positron-emission tomography
MRI: Magnetic resonance imaging
